# *KAMO*: towards automated data processing for microcrystals

**DOI:** 10.1107/S2059798318004576

**Published:** 2018-04-24

**Authors:** Keitaro Yamashita, Kunio Hirata, Masaki Yamamoto

**Affiliations:** a RIKEN SPring-8 Center, Sayo 679-5148, Japan

**Keywords:** automatic data processing, microcrystals, *KAMO*, small-wedge data sets

## Abstract

In protein microcrystallography, radiation damage often hampers complete and high-resolution data collection from a single crystal, even under cryogenic conditions. One promising solution is to collect small wedges of data (5–10°) separately from multiple crystals. The data from these crystals can then be merged into a complete reflection-intensity set. However, data processing of multiple small-wedge data sets is challenging. Here, a new open-source data-processing pipeline, *KAMO*, which utilizes existing programs, including the *XDS* and *CCP*4 packages, has been developed to automate whole data-processing tasks in the case of multiple small-wedge data sets. Firstly, *KAMO* processes individual data sets and collates those indexed with equivalent unit-cell parameters. The space group is then chosen and any indexing ambiguity is resolved. Finally, clustering is performed, followed by merging with outlier rejections, and a report is subsequently created. Using synthetic and several real-world data sets collected from hundreds of crystals, it was demonstrated that merged structure-factor amplitudes can be obtained in a largely automated manner using *KAMO*, which greatly facilitated the structure analyses of challenging targets that only produced microcrystals.

## Abstract 2

An automated data-processing pipeline for protein microcrystals is presented. The processing of multiple small-wedge data sets was made dramatically easier by this pipeline.

## 1. Introduction   

The major difficulty in solving protein structures by X-ray crystallography is obtaining crystals with suitable size and sufficient diffracting power. Recently, X-ray microbeams have become available at synchrotron light sources and have enabled data collection from microcrystals at high signal-to-noise ratios (Smith *et al.*, 2012 ▸; Owen *et al.*, 2016 ▸; Yamamoto *et al.*, 2017 ▸). The preparation of microcrystals is often easier than that of larger crystals, and smaller crystals may sometimes be better ordered and thus lead to higher quality data sets (Cusack *et al.*, 1998 ▸; Evans *et al.*, 2011 ▸). The lipidic mesophase method has facilitated the production of small but high-quality crystals of membrane proteins, which are typically difficult targets in protein crystallography (Caffrey, 2015 ▸). However, the radiation-dose limit of greater than 10 MGy is rapidly exceeded in microcrystals, and consequently multiple crystals are needed to overcome this problem. Serial femtosecond crystallography (SFX) using an X-ray free-electron laser (XFEL; Schlichting, 2015 ▸) is a very powerful approach in microcrystallography, based on the principle of ‘diffraction before destruction’. Since an ultra-intense femtosecond XFEL pulse only permits the collection of a single still diffraction image, thousands to tens of thousands of diffraction patterns are usually required for structural analysis. Although data collection from such a large number of crystals is fast at a high-repetition-rate XFEL, limited beamtime prevents its routine use. In contrast, radiation damage from synchrotron radiation cannot be completely avoided but high-resolution diffraction images can be collected *via* the rotation method, where integrated Bragg intensities are experimentally obtained. At cryogenic temperatures, small-wedge (5–10°) data collection can be a good compromise to obtain strong diffraction signals under tolerable doses, and tens to hundreds of data sets are usually sufficient to obtain a high-resolution structure. Efficient data collection can be achieved and easily automated when multiple microcrystals are held in a sample holder. *MeshAndCollect*, an automated multi-crystal data-collection workflow, has been developed at the ESRF (Zander *et al.*, 2015 ▸). The recently developed *ZOO* system (Hirata *et al.*, manuscript in preparation) at the microfocus beamline BL32XU at SPring-8 also supports the automatic collection of small-wedge data sets from loop-harvested microcrystals. These systems enabled rapid data collection from a large number of crystals. Multi-crystal data-collection strategies are advantageous over single-crystal data collection in obtaining accurate data which allow SAD phasing when correctly processed (Akey *et al.*, 2014 ▸; Liu & Hendrickson, 2015 ▸; Huang *et al.*, 2016 ▸; Olieric *et al.*, 2016 ▸). Small-wedge data collection is also compatible with room-temperature experiments that utilize crystallization plates, without the need for tedious crystal harvesting and cooling, as well as possibly allowing the exploration of functionally relevant protein conformations (Fraser *et al.*, 2011 ▸).

In small-wedge data collection, data processing is more complicated than processing single-crystal data. Although the indexing and integration of individual wedges may be straightforward, there are some potential issues to take note of, some of which are now listed: (i) space-group determination can be challenging for novel structure determination and any existing indexing ambiguity (Brehm & Diederichs, 2014 ▸) must be resolved, (ii) clustering may also be required to identify non-isomorphous data sets prior to merging and (iii) during merging, rogue data sets that deteriorate the overall data quality should be detected and removed. To help to address the aforementioned issues, several key technologies have been developed, including automated data-processing pipelines that process individual wedges, such as *xia*2 (Winter, 2010 ▸) and *autoPROC* (Vonrhein *et al.*, 2011 ▸). The optimized use of the *XDS* package for processing *in situ* diffraction data sets has been described previously (Huang *et al.*, 2015 ▸) and methods to resolve the indexing ambiguity have been developed for SFX (Brehm & Diederichs, 2014 ▸; Kabsch, 2014 ▸). Regarding clustering, two major methods have been presented, one based on the similarity of unit-cell parameters (Foadi *et al.*, 2013 ▸) and the other on the correlation coefficients of intensities (Giordano *et al.*, 2012 ▸). Yet another correlation-based approach that performs a multidimensional scaling analysis to sort out data by systematic differences has also been suggested (Diederichs, 2017 ▸). As the selection of isomorphous data is a combinatorial problem, the use of a genetic algorithm has been proposed (Zander *et al.*, 2016 ▸). To identify rogue data sets, a method based on CC_1/2_ has been presented (Assmann *et al.*, 2016 ▸). Despite the welcome advances in technology, the workflow as a whole entity has yet to be automated to the best of our knowledge, which is important for high-throughput analysis and should vastly aid decision making during beamline experiments.

Presented here is a new data-processing pipeline, *KAMO* (which in Japanese means a mallard, *Anas platyrhynchos*), which has been developed to automate the whole data-processing procedure for multiple small-wedge data sets by using existing programs including *XDS* (Kabsch, 2010 ▸), *DIALS* (Winter *et al.*, 2018 ▸) and *CCP*4 (Winn *et al.*, 2011 ▸). *KAMO* was originally developed for online use at the SPring-8 beamlines, but is now publicly available under an open-source license and can be used anywhere for small-wedge data sets. *KAMO* has already been used in several structure determinations from multiple microcrystals (Abe *et al.*, 2017 ▸; Shihoya *et al.*, 2017 ▸; Taniguchi *et al.*, 2017 ▸; Lee *et al.*, 2017 ▸; Miyauchi *et al.*, 2017 ▸; Suno *et al.*, 2017 ▸). In this article, the details of *KAMO* are described, followed by processing examples using synthetic and real-world data sets for the mercury-bound M_2_ receptor and cypovirus polyhedra, whereby the merged structure-factor amplitudes can be obtained with minimal user intervention using *KAMO*.

## 2. *KAMO* functions for multiple small-wedge data   

### 2.1. Program overview   

**Figure 1:**
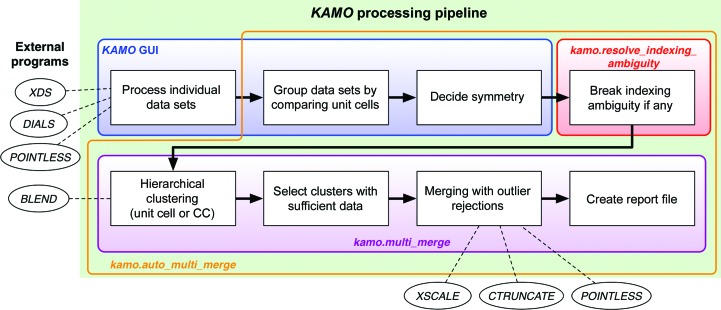
The workflow of *KAMO* for multiple small-wedge data sets with the program name for each role provided. The external programs that are required are also shown.

*KAMO* has been developed to automate the data processing of multiple wedges and merging. For this purpose, the processing of individual data sets, the selection of data sets belonging to the same lattice, clustering, merging with outlier rejections and the creation of a report were implemented. The workflow of *KAMO* is shown in Fig. 1 ▸. *KAMO* functionality was implemented in the *yamtbx* package (https://github.com/keitaroyam/yamtbx), which depends on the *cctbx* library (Grosse-Kunstleve *et al.*, 2002 ▸). The header information of several diffraction-image formats is obtained using a modified version of the *XIO* module originally included in the *xdsme* package (P. Legrand; https://github.com/legrandp/xdsme). Other dependent Python libraries include networkx for graph algorithms (Hagberg *et al.*, 2008 ▸) and SciPy for optimization and clustering (Jones *et al.*, 2001 ▸). The *XDS* package (Kabsch, 2010 ▸) is used for data processing: *XDS* for the indexing and integration of wedges and *XSCALE* for scaling and merging. From the *CCP*4 program suite (Winn *et al.*, 2011 ▸), *POINTLESS* (Evans, 2011 ▸), *CTRUNCATE* and *BLEND* (Foadi *et al.*, 2013 ▸) are used. *DIALS* (Winter *et al.*, 2018 ▸) is optionally supported for indexing and integration.

*KAMO* was originally developed for use at the SPring-8 beamlines, especially with the *ZOO* system. In the online mode, *KAMO* initiates data processing once all image files have become available by monitoring the log files of *BSS*, the standard data-collection program at SPring-8 (Ueno *et al.*, 2005 ▸). For offline use, the bl=other option can be specified to find data sets from the filesystem.

### 2.2. Data processing of individual wedges   

The processing of individual wedges is controlled through a GUI. When the kamo command is started, the files in the subdirectories are identified and by default the processing results are saved in the _kamoproc/ directory. The jobs are run in parallel using a queuing system (the Sun Grid Engine is currently supported) or on a local computer.

Indexing, integration and non-empirical intensity corrections are performed using *XDS* by default. In the preparation of XDS.INP, instrumental geometries, including the incident beam, goniometer and detector, are recognized from the image file header in the same way as for generate_XDS.INP (https://strucbio.biologie.uni-konstanz.de/xdswiki/index.php/Generate_XDS.INP). There is an option to use *dxtbx* (Parkhurst *et al.*, 2014 ▸) as implemented in *cctbx*, which may be more generic. *KAMO* does not usually assume that the unit-cell parameters and space-group symmetry are known *a priori*. Indexing is performed without any prior knowledge and integration is performed with *P*1 symmetry.

Known unit-cell parameters can optionally be used. In this case, they are used in indexing if an attempt without this information has failed. The integration will not be performed if the indexing result is inconsistent with the known unit cell. Providing known unit-cell parameters that are slightly incorrect can sometimes lead to an improper indexing result and users should be careful when using this option.

Scaling (JOB=CORRECT) is performed based on the symmetry deduced by *POINTLESS* (Evans, 2011 ▸). Non-empirical corrections, that is polarization and absorption by air and the sensor, are only applied in the CORRECT job with the CORRECTIONS= option, and the corrected intensity set is saved as XDS_ASCII.HKL_noscale. This file is used in merging where empirical corrections are performed. *POINTLESS* is used again for the XDS_ASCII.HKL file, and if the estimated Laue symmetry differs from that estimated from INTEGRATE.HKL, the candidate with higher probability is accepted. However, symmetry estimation may be less reliable in small-wedge cases because of the limited coverage of reciprocal space, and final symmetry determination for merging is deferred until all wedges are processed.

When *DIALS* is selected, *dials.index* is executed several times until success is obtained by trying all indexing methods with and without the local indexing option. Finally, the integration result is converted to the XDS_ASCII.HKL format and saved as DIALS.HKL.

**Figure 2:**
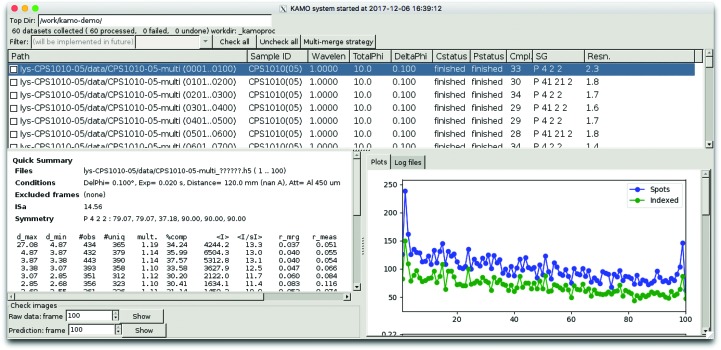
The *KAMO* GUI for processing individual wedges and for initiating the merging procedure. The collected data sets are listed with data-collection parameters and data-processing summaries. In the lower panel, the processing details for a selected item can be seen, including log files and plots with respect to image numbers.

The processing status of individual wedges is displayed on the GUI (Fig. 2 ▸). In the top half, wedges are listed with rotation ranges, processing success or failure, estimated space group and resolution. The details of the processing results are shown below by clicking the list, including the statistics table, plots and log files. In small-wedge cases, the statistics for individual wedges are not very informative and users should proceed to merging without trying to select wedges based on the statistics.

### 2.3. Preparation for merging   

#### 2.3.1. Grouping results by lattice and deciding on the space group   

**Figure 3:**
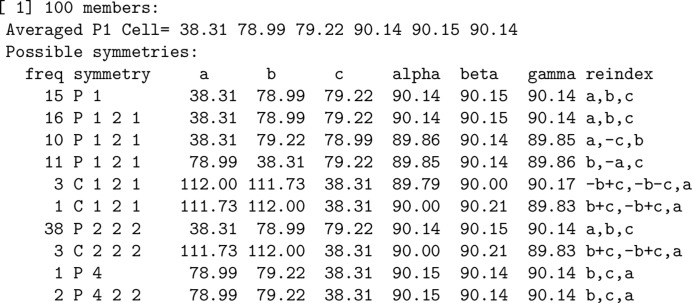
Grouping of indexed results based on unit-cell parameters in preparation for merging (titin data; simulated 1g1c). In this case all 100 data sets were indexed with a consistent unit cell. The averaged cell in *P*1 is shown and the possible point-group symmetries are listed with the ‘frequency’ of how many times *POINTLESS* assigned the symmetry.

Unit-cell parameters in *P*1 symmetry (Niggli reduced cell) of all data sets are initially compared with each other by taking possible reindexing operators into account. An undirected graph is constructed where each node represents a data set and an edge is drawn if two unit-cell parameters are sufficiently similar. The connected components are extracted from the graph and sorted by population. Small connected components are usually ignored because in most cases such data sets are wrongly indexed. For each group, possible point-group symmetries are listed, which are calculated based on the averaged unit-cell parameter (Zwart *et al.*, 2006 ▸). To help to decide the symmetry, the ‘frequencies’ (how often each symmetry was assigned by *POINTLESS*) are provided (Fig. 3 ▸). In our test cases, the most frequent symmetry was usually the correct one. Alternatively, users can assume a lower symmetry down to *P*1, break indexing ambiguities and let *POINTLESS* decide the symmetry using the scaled and merged results. The automated symmetry-determination procedure will be implemented in the future.

When the symmetry has been determined, the XDS_ASCII.HKL_noscale files (see §[Sec sec2.2]2.2) are reindexed to the specified unit-cell parameters and symmetry. The unit-cell parameters can optionally be refined by *XDS* (JOB=CORRECT) with constraints imposed by the selected Laue symmetry. New XDS_ASCII.HKL_noscale files are saved in the working directory for merging.

#### 2.3.2. Breaking indexing ambiguity   

There is ambiguity in the manner of indexing when the selected symmetry is lower than the highest possible symmetry. This always happens in point groups 23, 6, 32, 3 and 4, but also happens in, for example, point groups 222 when *a* ≃ *b* and 2 when β ≃ 90°, as in (pseudo)merohedral twinning. The indexing modes need to be made consistent among all files by reindexing each data set appropriately. The simplest way to achieve this is to use isomorphous reference data, which have common reflections with all data sets. Without an external reference, one of the data sets may be used as a reference data set provided that it shares enough reflections with the others, but this may not be the case for small-wedge data because the number of reflections in each data set is limited. The situation is similar to serial crystallography, where this problem is well known as the ‘indexing ambiguity’ problem. There are a number of solutions, including the Brehm and Diederichs algorithm (Brehm & Diederichs, 2014 ▸) and the selective breeding algorithm (Kabsch, 2014 ▸). The selective-breeding algorithm is implemented in *yamtbx* and is adopted as the default in *kamo.resolve_indexing_ambiguity* when an external isomorphous data set does not exist; otherwise the reference-based method can also be used in this command.

### 2.4. *kamo.multi_merge*: clustering and merging   

After preparing data sets with similar unit-cell parameters by a consistent indexing mode, clustering and merging with outlier rejections can be initiated using *kamo.multi_merge*. There are two options in hierarchical clustering: the unit cell-based method using *BLEND* functions (Foadi *et al.*, 2013 ▸) and the correlation coefficient (CC)-based method. The clusters are sorted by completeness and redundancy, and those that exceed a threshold (≥90% and ≥2 by default, respectively) are subjected to merging and outlier-rejection cycles. The GUI prepares shell scripts for the two clustering methods, which can easily be edited. Prior to the clustering analysis, data sets that have unit-cell parameters that are extremely different from the median values can be removed.

The CC-based clustering method uses a correlation coefficient of intensity that is calculated using the common reflections. *d*(*i*, *j*) = [1 − CC(*i*, *j*)]^1/2^ is used as the distance between data sets *i* and *j* and hierarchical clustering analysis is performed with the Ward method (Murtagh & Legendre, 2014 ▸). [1 − CC^2^(*i*, *j*)]^1/2^ can optionally be selected as used in Giordano *et al.* (2012 ▸). Data sets that have too few (<3 by default) reflections in common with any other data set are removed before clustering. This threshold can be increased by specifying the cc_clustering.min_common_refs option, which may stabilize CC calculation but reject more data sets. Data sets may have different intensity falloffs with respect to resolution, *i.e.* different overall *B* values, which invalidates the calculation of CC over whole reflections. To mitigate this problem, there is an option to use intensities corrected using Wilson *B* values or to use squared normalized structure-factor amplitudes |*E*|^2^.

For each cluster, data sets belonging to a cluster are subjected to merging with outlier rejections. *XSCALE* is used for merging and outliers are detected by analyzing the *XSCALE* output. There are two kinds of outliers that should be removed, bad frames and bad crystals, both of which deteriorate the merging results. Bad frames may be identified when the crystal is out of the beam (often the first and last frames when incorrectly centred) or show decay owing to radiation damage, which is often the case in room-temperature experiments. Bad crystals (data sets) may be attributed to, for example, poorly performed integration with incorrect predictions and poor isomorphism. The merging and outlier-rejection procedure is performed for up to three cycles. Initially, all data sets in the cluster are scaled and merged and bad frames are detected by comparing the intensities of each frame against the merged data. The bad frames are identified by a CC that is too small based on Tukey’s 1.5 × IQR criterion (Tukey, 1977 ▸) or a user-specified value. Secondly, bad data sets are detected from the merged result excluding bad frames. Several detection methods have been implemented using the error-model parameters *ab* or *b*, the scaling parameter *B* and the *R* factor. The error-model parameters *a* and *b* are refined for each data set in *XSCALE* to correct the error of intensity σ(*I*) through σ^2^(*I*) = *a*[σ^2^_counting_(*I*) + *bI* ^2^], and large *b* or *ab* values indicate that the data set has large systematic errors (Diederichs, 2010 ▸). By default, data sets with extreme values of *b* or *B* are removed. The remaining data sets are merged using the data set with the smallest intensity falloff with respect to resolution (the smallest *B* in *XSCALE*) as a scaling reference. However, in the first cycles a data set with median *B* is used as a reference because using a data set with an extremely small *B* as a reference sometimes results in useless statistics and merged data. This is probably because such data sets at extrema tend to be outliers and scaling against them can degrade scaling coefficients. The scaled intensity is converted to MTZ format using *XDSCONV* and analyzed using *phenix.xtriage* (Zwart *et al.*, 2005 ▸) and *CTRUNCATE* (Winn *et al.*, 2011 ▸). The MULTIPLICITY column is added to the MTZ file to check the multiplicity of each reflection. Diffraction anisotropy is analyzed with weighted CC_1/2_ along the principal axes using the same method as in *AIMLESS* (Evans & Murshudov, 2013 ▸).

In *XSCALE*, three kinds of corrections are performed: decay, modulation and absorption. The NBATCH= parameter, which defines the number of scaling batches, is automatically determined by *XSCALE* by default; however, when severe radiation damage occurs it may help to increase NBATCH=. In *kamo.multi_merge*, the number of frames per scaling batch can be controlled using either the xscale.frames_per_batch or xscale.degrees_per_batch parameter, and a small number (for example one) should be given if radiation damage is severe.

The summary and details of clustering and merging results are reported in an HTML file with visualization using amCharts and D3.js (Supplementary Fig. S1).

### 2.5. *kamo.auto_multi_merge*: fully automated merging for multiple samples   

*kamo.multi_merge*, as described above, is useful when processing data in a semi-automated manner by manually setting parameters to optimize the results. When users want to process many data sets of multiple samples at the same time in a fully automated manner, *kamo.auto_multi_merge* can be used. This program finds processed results from the *KAMO* GUI, deduces the space group, breaks the indexing ambiguity, runs *kamo.multi_merge*, determines the high-resolution cutoff for each sample and runs *kamo.multi_merge* once more with this cutoff. The high-resolution cutoff is determined by fitting the curve of CC_1/2_ *versus* resolution shells as used in *AIMLESS* and undergoes fine-tuning to ensure that the outer-shell CC_1/2_ is close to the specified value (0.5 by default). The result with the highest overall and outer-shell CC_1/2_ is considered to be the best result. Sample information can be prepared using a CSV file, where the root directory, sample names, anomalous flag and reference data file (optional) are specified. The report is an HTML file, which is generated to review all merging results at the same time.

### 2.6. Availability   

The source code of *KAMO* is available under the new BSD license at GitHub (https://github.com/keitaroyam/yamtbx). Documentation, including installation notes, is available at https://github.com/keitaroyam/yamtbx/blob/master/doc/kamo-en.md. Installation is easy when *PHENIX* or *DIALS* is already installed because they include most of the dependencies, including *cctbx* and wxPython.

## 3. Examples   

*KAMO* has been used for several structure analyses (Table 1 ▸). Processing notes describing the analysis of raw data that are publicly available can be found at https://github.com/keitaroyam/yamtbx/wiki. In this section, some processing examples are presented using the methods described here. The program versions used here were *yamtbx* commit 1107fc4 (as of 26 December 2017) with *cctbx* commit 16b2e7c (as of 17 April 2017); *XDS* 1 May 2016 (BUILT=20160617); *CCP*4 7.0 (update 042); *PHENIX* 1.11.1; *SHELXC*, *SHELXD* and *SHELXE* 2016/1, 2013/2 and 2016/3, respectively; and *R* 3.2.2. For data processing using *XDS*, diffraction images in the HDF5 format were read through the modified version of Takanori Nakane’s *eiger*2*cbf* (https://github.com/keitaroyam/eiger2cbf). The processing results may differ slightly from the original studies because of the use of different versions.

All data sets except for the synthetic data were collected at BL32XU, SPring-8, Hyogo, Japan.

### 3.1. James Holton’s microfocus data-processing challenge   

James Holton opened ‘The Micro-focus Data Processing Challenge’ (http://bl831.als.lbl.gov/~jamesh/challenge/microfocus) using simulated diffraction images of titin (PDB entry 1g1c) with an anomalous signal from selenomethionine at 0.9793 Å wavelength. We downloaded the minimal version of the data, which consisted of 100 wedges of 3°. These synthetic data have two issues: severe radiation damage and indexing ambiguity caused by the space group, *P*2_1_2_1_2_1_ with *b* ≃ *c* (*a* = 38.3, *b* = 78.6, *c* = 79.6 Å).

**Figure 4:**
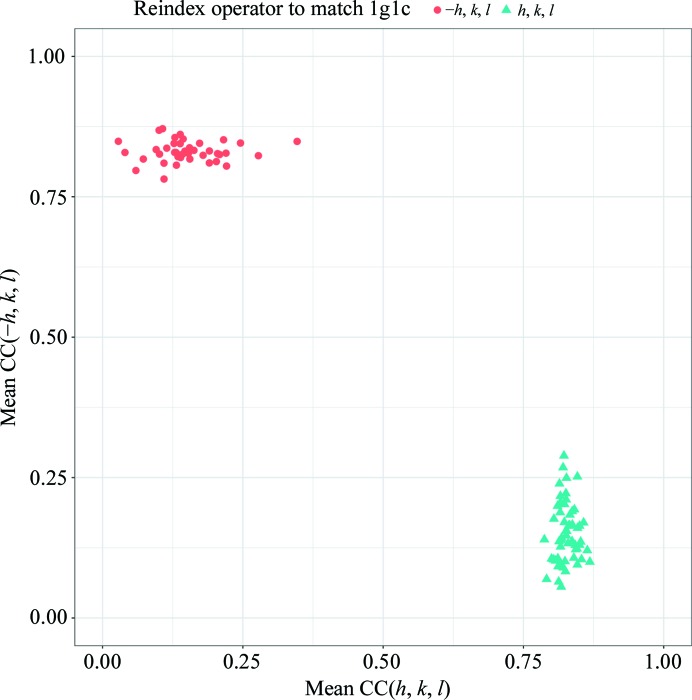
Breaking indexing ambiguity in the titin data (simulated 1g1c). The averaged CC with all other wedges in the final (converged) cycle is plotted for two possible modes (*h*, *k*, *l* and −*h*, *l*, *k*). The resolution was validated using the original 1g1c data (shown as symbols and colours). This figure was prepared using *ggplot*2 (Wickham, 2009 ▸) in *R* (R Development Core Team, 2008 ▸).

**Figure 5:**
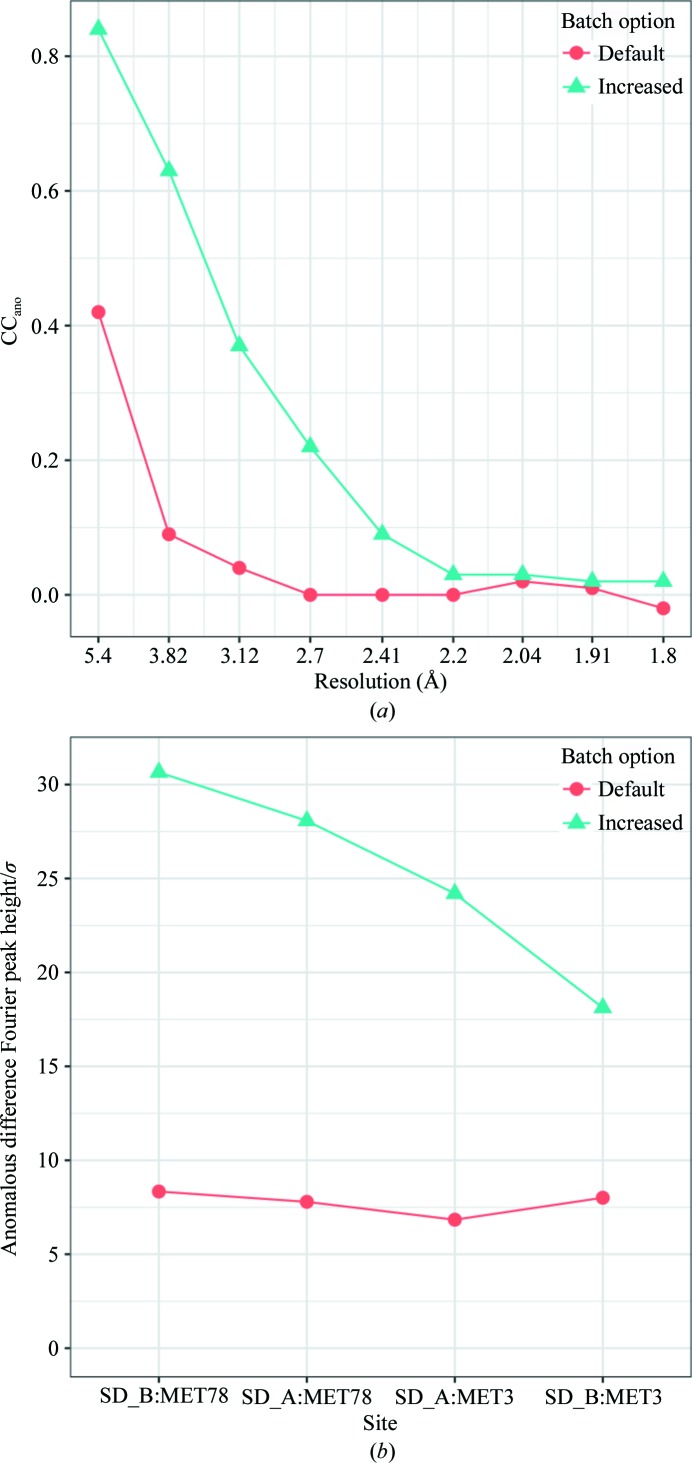
Improved anomalous data quality by increasing the scaling batches in the titin data (simulated 1g1c). To increase the number of batches the option xscale.degrees_per_batch=1 was given in *kamo.multi_merge*. (*a*) CC_ano_ is the correlation coefficient of *I* ^(+)^ − *I* ^(−)^ between random half-sets. (*b*) Anomalous difference Fourier peak heights calculated using the observed anomalous differences and the 1g1c model with *SHELXC* and *ANODE* (Thorn & Sheldrick, 2011 ▸).

Here, we demonstrate automatic data processing, space-group determination, resolution of indexing ambiguity and merging using *KAMO* followed by successful SAD phasing. The *KAMO* GUI was started with default parameters and all 100 data sets were processed by *XDS* without failure. The most frequent point-group symmetry determined by *POINTLESS* for each data set was *P*222 (38 times; Fig. 3 ▸). All integration results were reindexed to *P*222 symmetry. The indexing ambiguity was resolved by *kamo.resolve_indexing_ambiguity* with the selective breeding algorithm in two cycles, which was later validated using the 1g1c data (Fig. 4 ▸). *kamo.multi_merge* was then used for clustering and merging with outlier rejections at 1.8 Å resolution (the detector edge). Clustering was found to be unnecessary because the largest cluster (using all data) resulted in the best statistics and phase quality. As radiation damage was severe, the option xscale.degrees_per_batch=1 to increase the number of scaling batches so that one batch corresponded to 1° was essential for the success of SAD phasing, and also gave better data statistics and higher anomalous difference Fourier peak heights (Fig. 5 ▸). The data-processing, phasing and refinement statistics with a comparison with the non-*KAMO* results are summarized in Supplementary Table S1. Determination of the heavy-atom sites, phasing and phase improvement were performed with *SHELXC*/*D*/*E* (Sheldrick, 2010 ▸). All four heavy-atom sites were correctly located by *SHELXD* and 157 polyalanine residues were built by *SHELXE* in four cycles. The resulting CC against the native data was 39.45%.

### 3.2. M_2_ receptor: Hg-SAD phasing   

Here, a real SAD phasing case is demonstrated using the human muscarinic acetylcholine receptor M_2_ (M_2_R). 671 small-wedge (5° per crystal) data sets were collected from mercury-bound M_2_R-BRIL crystals (PDB entry 5yc8) using the *ZOO* system and an MX225-HS CCD detector (Rayonix) to obtain model bias-free phase information by SAD phasing (Suno *et al.*, manuscript in preparation). Exposure conditions were automatically adjusted so that each crystal absorbed 7–12 MGy. The expected anomalous signal 〈|Δ*F*_ano_|〉/〈|*F*|〉 was 4.2% at 1 Å wavelength with full occupancies, which was calculated using the equation of Hendrickson & Teeter (1981 ▸). The actual anomalous signal was estimated to be 3.2% based on the *F*_model_ of the refined structure. The raw diffraction images will be made available in the Zenodo data repository (https://doi.org/10.5281/zenodo.1172266) when the associated manuscript is published.

The *KAMO* GUI was started with default parameters, and 547 data sets were indexed and integrated using *XDS*. Of these, 532 data sets had consistent unit-cell parameters, with the most frequent (341 times) point-group symmetry being *P*2; the data sets were thus reindexed in *P*2. Clustering by unit-cell parameters was performed using *BLEND*. SAD phasing was performed with the *SHELXC*/*D*/*E* programs (Sheldrick, 2010 ▸). 10 000 trials to find three mercury sites were performed using *SHELXD* and 30 cycles of polyalanine tracing after phase improvement assuming 55% solvent content were performed with *SHELXE*. From the largest cluster consisting of all data sets, 238 polyalanine residues were built with a CC of 39.03% by *SHELXE* in 24 cycles and the electron-density map was sufficiently clear to trace the M_2_R structure. The data processing, phasing and refinement statistics with a comparison with the non-*KAMO* results are summarized in Supplementary Table S2.

To compare clusters, the real-space CC of the SAD-phased electron-density map against the map calculated from the 5yc8 model (CC_map_) was calculated for each cluster using *phenix.get_cc_mtz_pdb* (Adams *et al.*, 2010 ▸). The correctness of the located Hg sites and CC_map_ are summarized in Table 2 ▸ along with representative statistics and unit-cell variations. The largest cluster had the best phase quality despite large unit-cell variations. CC-based clustering was also attempted; however, no subcluster offering better quality was found.

### 3.3. Cypovirus polyhedra: clustering   

Here, the power of clustering to achieve a better result is demonstrated using cypovirus polyhedra, a natural crystalline protein assembly of the polyhedrin monomer produced in infected insect cells (Abe *et al.*, 2017 ▸). 184 small-wedge (5°) data sets for a deletion mutant Δ3 (PDB entry 5gqn) were collected automatically using *ZOO* and an EIGER X 9M detector (DECTRIS). Exposure conditions were automatically adjusted so that each crystal absorbed 11.6 MGy. The crystals belonged to space group *I*23, with unit-cell parameter *a* ≃ 103 Å. The raw diffraction images are available from the Zenodo data repository (https://doi.org/10.5281/zenodo.846473).

The *KAMO* GUI was started with default parameters and 157 data sets were successfully indexed and integrated with a consistent unit cell. The most frequent (122 times) symmetry was *I*23, which was consistent with the known structure (PDB entry 2oh6; Coulibaly *et al.*, 2007 ▸). The indexing ambiguity was resolved using the known isomorphous reference data (PDB entry 2oh6) by *kamo.resolve_indexing_ambiguity*.

For merging, *kamo.multi_merge* was used to 1.55 Å resolution and several clustering metrics, *BLEND*, CC(*I*) and CC(|*E*|^2^), were compared. Structure refinement was performed for all merged results using *phenix.refine* starting from the 5gqn model with strategies of rigid-body, individual ADP and coordinate, and occupancy refinement. The top 12 results were sorted according to *R*_free_ and are listed in Table 3 ▸. CC(|*E*|^2^) gave the best cluster (multiplicity of 9.7) in CC_1/2_ and *R*_free_ rather than merging all data sets even after outlier rejections (multiplicity of 67.8). The data-processing and refinement statistics with a comparison with the non-*KAMO* results are summarized in Supplementary Table S3.

The reason why clustering was not useful in the other data sets mentioned above may be partly attributable to the limited number of common reflections for the calculation of CC owing to lower space-group symmetries. Moreover, the accuracy of subclusters may be compromised because of insufficient multiplicity when the total amount of data is limited. However, if almost all crystals are isomorphous, merging more data sets would simply improve the data quality and resolution.

## 4. Conclusions and outlook   

*KAMO*, an automated data-processing system for multiple microcrystals, has been developed and its use is spreading in facilities across the world. *KAMO* dramatically simplifies the processing of multiple small-wedge data sets, working with *XDS* and *CCP*4 programs. Data collection from a number of crystals was highly beneficial for successful SAD phasing (§[Sec sec3.2]3.2) and finding the high-quality subset of the whole data (§[Sec sec3.3]3.3), which were easily achieved through *KAMO*.

This system has been routinely used on BL32XU and often combined with the automated data-collection system *ZOO*, which enables a quick evaluation of the collected data sets. These systems enable high-throughput and high-resolution structure analyses of challenging targets, crystals of which are often difficult to grow to large sizes (Abe *et al.*, 2017 ▸; Shihoya *et al.*, 2017 ▸; Taniguchi *et al.*, 2017 ▸; Lee *et al.*, 2017 ▸; Miyauchi *et al.*, 2017 ▸; Suno *et al.*, 2017 ▸). In many cases, the default parameters of *KAMO* are adjusted to result in sufficient data quality, but some fine-tuning may be required to obtain better quality, such as sampling several clustering methods. Future directions include better algorithms for clustering and outlier rejections, full automation without any human intervention, implementation of all functions in the GUI and on-the-fly merging functions. On-the-fly merging will provide the resolution and data quality of the collected data sets, and improve efficiency by aiding decision making on whether to stop the experiment and switch to another sample.

## Supplementary Material

Supplementary Tables and Figures.. DOI: 10.1107/S2059798318004576/wa5117sup1.pdf

## Figures and Tables

**Table 1:** Current structure analyses that *KAMO* has contributed to

Sample	Polyhedra† (wild type)	Polyhedra† (mutant)	LPA_6_ ‡	ET_B_R§	TPT¶ (P_i_)	TPT¶ (3-PGA)	AtDTX14††	OX2R‡‡
PDB code	5gqm	5gqn	5xsz	5xpr	5y78	5y79	5y50	5wqc
Resolution (Å)	1.68	1.55	3.2	3.5	2.1	2.2	2.6	1.96
Space group	*I*23	*I*23	*P*2_1_2_1_2_1_	*P*3_2_21	*P*2_1_2_1_2	*P*2_1_2_1_2	*P*2_1_2_1_2_1_	*C*2
Degrees per data set	5	5	4, 6	10	3–10, 30	3–10	5, 10, 20	1–6
No. of data sets collected	20	184	397	16	723	332	373	805
No. of data sets processed for merging	20	155	350	16	599	250	139	768
No. of merged data sets	14	41	241	14	319	199	100	631

† Cypovirus polyhedra (Abe *et al.*, 2017 ▸).

‡ Lysophosphatidic acid receptor LPA_6_ (Taniguchi *et al.*, 2017 ▸).

§ Endothelin ET_B_ receptor bound to bosentan (Shihoya *et al.*, 2017 ▸).

¶ Triose-phosphate/phosphate translocator bound to P_i_ and 3-PGA (Lee *et al.*, 2017 ▸).

†† Eukaryotic MATE transporter AtDTX14 (Miyauchi *et al.*, 2017 ▸).

‡‡ Human orexin 2 receptor (Suno *et al.*, 2017 ▸).

**Table 2:** Data and SAD phase quality for clusters by unit-cell similarities using *BLEND* for mercury-bound M_2_R data (PDB entry 5yc8) The top eight results sorted by multiplicity are shown. LCV is the linear cell variation defined in *BLEND* (Foadi *et al.*, 2013 ▸). The inner and outer resolution ranges are 50–7.50 and 2.65–2.50 Å, respectively. The correctness of the located Hg sites was evaluated against those of the 5yc8 model using *phenix.emma* (Adams *et al.*, 2002 ▸). *B*_Wilson_ is the Wilson *B* value reported by *CTRUNCATE*. Completeness is greater than or equal to 98% for every case. For the calculation of multiplicity, CC_1/2_ and CC_ano_, Friedel pairs are treated as different reflections.

						Hg correctly located			
LCV (%)	Cluster height	No. of data sets merged	Multiplicity	CC_1/2_ (outer)	CC_ano_ (inner)	No.	R.m.s.d. (Å)	CC_map_	*B*_Wilson_ (Å^2^)
19.6	88.56	454	20.7	0.611	0.80	3	0.60	0.575	33.8
5.7	71.17	421	19.2	0.606	0.79	2	0.38	0.529	33.8
4.6	56.42	388	17.7	0.582	0.76	3	1.16	0.506	33.3
4.6	48.77	283	13.0	0.460	0.67	3	1.27	0.505	34.1
4.0	42.69	215	10.0	0.379	0.63	3	0.75	0.415	33.7
4.0	28.15	184	8.6	0.349	0.59	2	0.67	0.317	33.7
2.1	15.92	135	6.3	0.264	0.54	2	0.41	0.109	33.2
1.5	18.51	103	4.7	0.109	0.50	2	0.66	0.076	29.9

**Table 3:** Comparison of clustering methods using polyhedra data (PDB entry 5gqn) The top 12 results sorted by *R*_free_ are shown out of 99 clusters tested. The inner and outer resolution ranges are 50–4.65 and 1.65–1.55 Å, respectively. 〈*B*〉 is the averaged atomic *B* value reported by *phenix.refine*. Completeness is greater than or equal to 99.8% for every case.

Clustering method	LCV (%)	No. of data sets merged	Multiplicity	CC_1/2_ (inner)	CC_1/2_ (outer)	*R*_work_	*R*_free_	〈*B*〉 (Å^2^)
CC(|*E*|^2^)	0.4	18	9.7	0.989	0.753	0.1411	0.1765	10.7
CC(|*E*|^2^)	0.4	15	7.7	0.985	0.734	0.1436	0.1797	9.7
CC(|*E*|^2^)	0.9	24	13.1	0.941	0.805	0.1443	0.1807	10.5
CC(*I*)	0.4	29	15.5	0.988	0.666	0.1434	0.1816	10.4
CC(*I*)	0.9	38	19.9	0.989	0.670	0.1447	0.1823	9.7
CC(*I*)	0.4	22	11.9	0.983	0.627	0.1437	0.1825	10.6
CC(*I*)	0.4	17	8.7	0.980	0.620	0.1459	0.1828	10.4
CC(*I*)	0.4	18	9.2	0.978	0.596	0.1475	0.1839	9.2
CC(*I*)	0.4	25	13.2	0.984	0.647	0.1449	0.1841	10.2
CC(*I*)	2.2	96	49.0	0.986	0.726	0.1474	0.1849	8.8
*BLEND*	1.2	110	57.3	0.984	0.722	0.1481	0.1871	8.0
All data	2.2	130	67.8	0.969	0.712	0.1551	0.1939	7.1
