# Supplementary material for: KAMO: towards automated data processing for microcrystals
Source: Acta Crystallogr D Struct Biol. 2018 Apr 24;74(Pt 5):441–9. doi: 10.1107/S2059798318004576 (PMC5930351; doi:10.1107/S2059798318004576)
Supplement: Supplementary file 1 [file d-74-00441-sup1.pdf]

STRUCTURAL BIOLOGY

Volume 74 (2018)

Supporting information for article:

KAMO: toward automated data processing for microcrystals

Keitaro Yamashita, Kunio Hirata and Masaki Yamamoto

See original cluster dendrogram

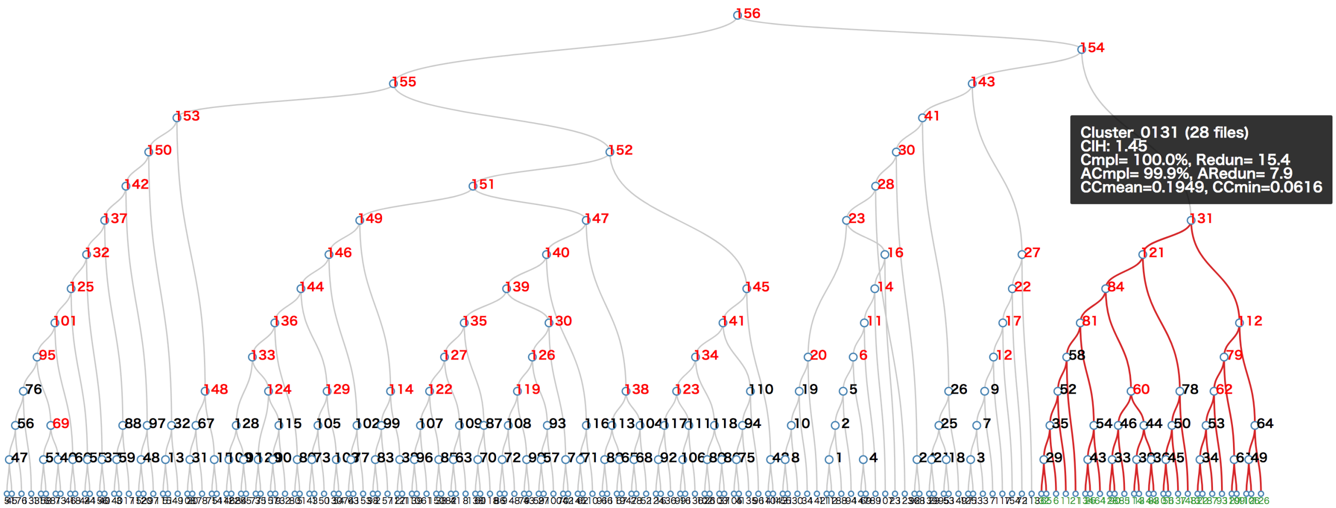

Show/Hide cluster list

Merging summary

	cluster	CIH	LCV	aLCV	#DS all	#DS used	Overall					Outer shell					Inner shell							B_{Wilson}	Aniso resol		d_{min} est.
							Cmpl	Redun	I/σ (I)	R_{meas}	$CC_{1/2}$	Cmpl	Redun	I/σ (I)	R_{meas}	$CC_{1/2}$	Cmpl	Redun	I/σ (I)	R_{meas}	$CC_{1/2}$	SigAno	CC_{ano}		best	wrst	
▼	cluster_0156	4.43	2.2	3.2	157	130	100.0	67.8	8.61	82.0	96.5	100.0	60.7	2.03	485.8	71.2	99.8	72.4	21.13	39.8	96.9	1.3	4.0	4.21	nan	nan	1.48
▼	cluster_0155	2.26	2.2	3.1	99	85	100.0	44.6	3.28	127.9	84.9	100.0	37.5	-99.00	-99.9	0.9	99.8	48.6	11.57	57.3	81.4	1.5	-4.0	6.36	nan	nan	1.97
▼	cluster_0152	1.75	2.1	3.1	71	61	99.9	32.0	3.24	122.2	85.5	99.9	27.4	0.29	1156.5	-0.4	99.8	34.8	10.79	51.0	85.2	1.4	-4.0	5.42	nan	nan	1.98
▲	cluster_0154	2.08	1.0	1.5	58	51	100.0	27.6	7.81	41.8	98.3	100.0	27.2	2.20	158.7	68.9	99.8	28.7	16.77	25.0	98.6	0.9	1.0	8.47	nan	nan	1.50

```
 SPACE_GROUP_NUMBER=  197
 UNIT_CELL_CONSTANTS=   103.38   103.38   103.38  90.000  90.000  90.000

 SUBSET OF INTENSITY DATA WITH SIGNAL/NOISE >= -3.0 AS FUNCTION OF RESOLUTION
 RESOLUTION     NUMBER OF REFLECTIONS    COMPLETENESS R-FACTOR  R-FACTOR COMPARED I/SIGMA   R-meas  CC(1/2)  Anomal  SigAno   Nano
   LIMIT     OBSERVED  UNIQUE  POSSIBLE     OF DATA   observed  expected                                      Corr

     4.65       30678    1069      1071       99.8%      24.6%     25.5%    30678   16.77     25.0%    98.6*     1    0.908     877
     3.29       47314    1847      1847      100.0%      28.8%     25.3%    47314   15.20     29.4%    98.2*    -3    0.985    1650
     2.68       67433    2391      2391      100.0%      29.4%     27.5%    67433   13.37     29.9%    97.8*    -4    0.927    2198
     2.33       78334    2714      2714      100.0%      33.5%     31.0%    78334   11.48     34.1%    97.2*    -2    0.929    2525
     2.08       87794    3192      3192      100.0%      40.4%     35.7%    87794    9.56     41.2%    96.2*    -2    0.928    2994
     1.90       90546    3441      3442      100.0%      54.9%     42.6%    90546    7.36     56.0%    94.6*    -3    0.908    3247
     1.76      103811    3711      3711      100.0%      65.9%     70.0%   103811    5.40     67.1%    90.8*    -4    0.827    3522
     1.64      121005    4268      4268      100.0%      98.3%    123.0%   121005    3.54    100.1%    83.3*     0    0.788    4063
     1.55      112740    4152      4152      100.0%     155.7%    214.8%   112740    2.20    158.7%    68.9*    -4    0.708    3965
    total      739655   26785     26788      100.0%      41.0%     40.3%   739655    7.81     41.8%    98.3*    -2    0.857   25041

Anisotropy:
 No anisotropy in this symmetry.
```

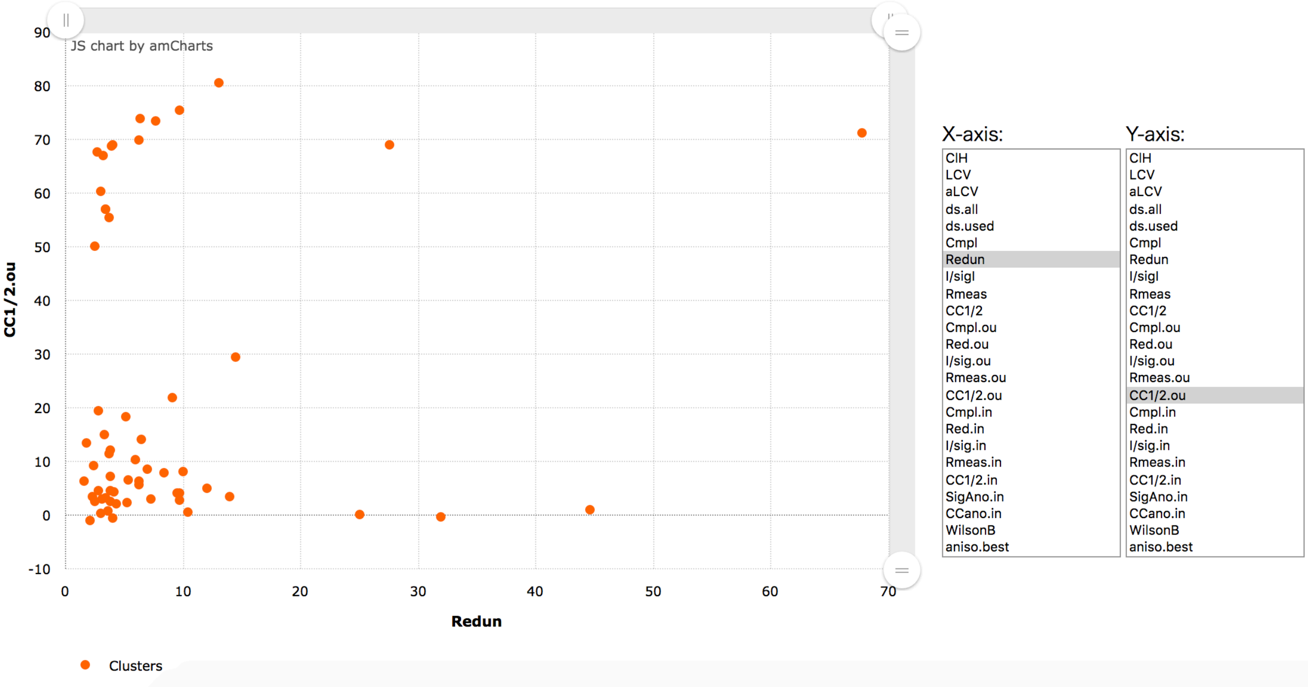

Fig. S1: A bottom part of a reported HTML file generated by *kamo.multi_merge* for polyhedra data (PDB code: 5GQN) clustered by CC($|E|^2$). Interactive cluster dendrogram (cluster highlighted on mouse-over), table of statistics for each cluster, and interactive scatter plot to compare clusters by statistics are shown. In the plot, $CC_{1/2}$ (outer) vs redundancy is shown in the figure.

Table S1: Data processing and refinement statistics with and without KAMO for titin data (simulated 1G1C)

	Non-KAMO (all data merged)			KAMO		
Data processing						
No. merged datasets	100			92		
Unit cell parameters (Å)	$a = 38.3, b = 79.1, c = 79.1$			$a = 38.3, b = 79.1, c = 79.1$		
LCV (%)	0.7			0.7		
	overall	inner shell	outer shell	overall	inner shell	outer shell
Resolution range (Å)	50–1.80	50–5.40	1.91–1.80	50–1.80	50–5.40	1.91–1.80
Completeness (%)	99.7	99.2	99.7	99.5	99.0	99.4
Multiplicity	6.0	6.1	5.9	5.4	5.6	5.3
R_{meas}	0.440	0.175	1.022	0.163	0.056	0.898
$\langle I/\sigma(I)\rangle$	2.99	6.61	1.02	11.69	27.53	3.45
$\text{CC}_{1/2}$	0.921	0.979	0.482	0.995	0.997	0.702
CC_{ano}	0.03	0.41	-0.02	0.19	0.84	0.02
ANODE max peak	8.99σ			30.65σ		
Refinement						
R_{work}	0.2450			0.2209		
R_{free}	0.2871			0.2516		
Average B values (Å^2)	19.9			20.1		
R.m.s.d. from ideal						
bond lengths (Å)	0.007			0.007		
bond angles (°)	0.873			0.903		
Ramachandran plot						
Favored (%)	100			100		
Allowed (%)	0			0		
Outlier (%)	0			0		
Phasing						
Substructure search (SHELXD)						
High-resolution cutoff (Å)	2.3			2.3		
CC_{all} (%)	12.89			41.91		
CC_{weak} (%)	8.60			26.88		
Phasing and DM (SHELXE)						
Polypeptide residues built	61			157		
CC against native data (%)	10.12			39.45		
CC_{map} (to 1G1C model)	0.018			0.697		

Friedel pairs are treated as different reflections. In both cases, KAMO was used to prepare files for merging. In non-KAMO just the XSCALE was run with default parameters using all prepared XDS_ASCII.HKL files without any clustering or rejections. 'KAMO' is the result obtained using *kamo.multi_merge* (with clustering and outlier rejections, and increased scaling batches).

Table S2: Data processing and refinement statistics with and without KAMO for mercury-bound M_2R data (5YC8)

	Non-KAMO (all data merged)			KAMO		
Data processing						
No. merged datasets	532			454		
Unit cell parameters (Å; °)	$a = 46.5, b = 59.0, c = 89.3; \beta = 98.9$			$a = 46.5, b = 59.0, c = 89.3; \beta = 98.9$		
LCV (%)	19.6			19.6		
	overall	inner shell	outer shell	overall	inner shell	outer shell
Resolution range (Å)	50–2.50	50–7.50	2.65–2.50	50–2.50	50–7.50	2.65–2.50
Completeness (%)	99.9	99.3	99.9	99.9	99.3	99.9
Multiplicity	25.8	26.1	24.9	20.7	20.8	20.1
R_{meas}	1.116	0.160	6.511	0.452	0.105	4.677
$\langle I/\sigma(I) \rangle$	9.60	35.87	1.41	9.48	35.59	1.39
$CC_{1/2}$	0.963	0.998	0.596	0.996	0.998	0.611
CC_{ano}	0.17	0.76	0.01	0.19	0.80	-0.02
ANODE max peak	26.0σ			26.7σ		
Refinement						
R_{work}	0.2364			0.2260		
R_{free}	0.2837			0.2748		
Average B values (Å^2)	43.6			64.2		
R.m.s.d. from ideal						
bond lengths (Å)	0.008			0.008		
bond angles (°)	0.890			0.883		
Ramachandran plot						
Favored (%)	97.09			97.09		
Allowed (%)	2.65			2.65		
Outlier (%)	0.26			0.26		
Phasing						
Substructure search (SHELXD)						
High-resolution cutoff (Å)	3.0			3.0		
CC_{all} (%)	28.57			34.09		
CC_{weak} (%)	16.74			19.36		
Phasing and DM (SHELXE)						
Polypeptide residues built	259			238		
CC against native data (%)	39.64			39.03		
CC_{map} (to 5YC8 model)	0.532			0.575		

Friedel pairs are treated as different reflections. In both cases, KAMO was used to prepare files for merging. In non-KAMO just the XSCALE was run with default parameters using all prepared XDS_ASCII.HKL files without any clustering or rejections. 'KAMO' is the result obtained using *kamo.multi_merge* (with clustering and outlier rejections).

Table S3: Data processing and refinement statistics with and without KAMO for polyhedra data (5GQN)

	Non-KAMO (all data merged)			KAMO		
Data processing						
No. merged datasets	157			18		
Unit cell parameter (Å)	$a = 103.6$			$a = 103.2$		
LCV (%)	2.2			0.4		
	overall	inner shell	outer shell	overall	inner shell	outer shell
Resolution range (Å)	50–1.55	50–4.65	1.64–1.55	50–1.55	50–4.65	1.64–1.55
Completeness (%)	100.0	99.8	100.0	99.9	99.7	99.9
Multiplicity	83.8	89.7	74.8	9.69	10.1	9.52
R_{meas}	1.799	0.452	5.778	0.274	0.140	0.896
$\langle I/\sigma(I)\rangle$	7.64	20.76	1.24	6.59	13.92	2.09
$\mathrm{CC}_{1/2}$	0.699	0.940	0.352	0.984	0.989	0.753
Refinement						
R_{work}	0.1981			0.1411		
R_{free}	0.2438			0.1765		
Average B values (Å^2)	7.1			10.7		
R.m.s. from ideal						
bond lengths (Å)	0.006			0.006		
bond angles (°)	0.833			0.869		
Ramachandran plot						
Favored (%)	96.28			96.69		
Allowed (%)	2.89			2.48		
Outlier (%)	0.83			0.83		

In both cases, KAMO was used to prepare files for merging. In non-KAMO just the XSCALE was run with default parameters using all prepared XDS_ASCII.HKL files without any clustering or rejections. 'KAMO' is the result obtained using *kamo.multi_merge* (with clustering and outlier rejections).
